# The effect of electron density in furan pendant group on thermal-reversible Diels–Alder reaction based self-healing properties of polymethacrylate derivatives

**DOI:** 10.1039/c8ra07268j

**Published:** 2018-11-26

**Authors:** Keum-Seob Byun, Won Jae Choi, Ha-Young Lee, Min-Ji Sim, Sang-Ho Cha, Jong-Chan Lee

**Affiliations:** Department of Chemical Engineering, Kyonggi University Suwon-Si 16227 South Korea sanghocha@kgu.ac.kr +82 31 257 0161 +82 31 249 9783; School of Chemical and Biological Engineering and Institute of Chemical Processes, Seoul National University 599 Gwanak-ro, Gwanak-gu Seoul 151-742 Republic of Korea jongchan@snu.ac.kr +82 2 880 8899 +82 2 880 7070

## Abstract

Herein, we discuss the effect of electron density in a furan pendant group on the thermally reversible Diels–Alder (DA) reaction based self-healing efficiency in polymethacrylate derivatives. First, the furan-functionalized polymethacrylates (rPFMA and dPFMA) having different electron density in the furan pendant groups were prepared through free-radical polymerization. The healing efficiency of rPFMA, which was expected to have high healing efficiency due to the high reactivity of DA reaction originating from the electron density in the furan moiety, was shown to be 95.89% in the first and 69.86% in the second healing process, respectively, where it is higher than that of dPFMA having relatively low electron density in the furan moiety. To illustrate these results, kinetic tests of the DA reaction for rPFMA64 and dPFMA64 were performed, where the reactivity of the DA reaction for rPFMA64 was much higher than that for dPFMA64. This could be explained by the electron density in the furan pendant groups which controls the reactivity of DA reaction having a major effect on the efficiency of self-healing performance in furan-functionalized polymethacrylates.

## Abstract 2

Herein, we discuss the effect of electron density in a furan pendant group on the thermally reversible Diels–Alder (DA) reaction based self-healing efficiency in polymethacrylate derivatives.

## Graphical abstract

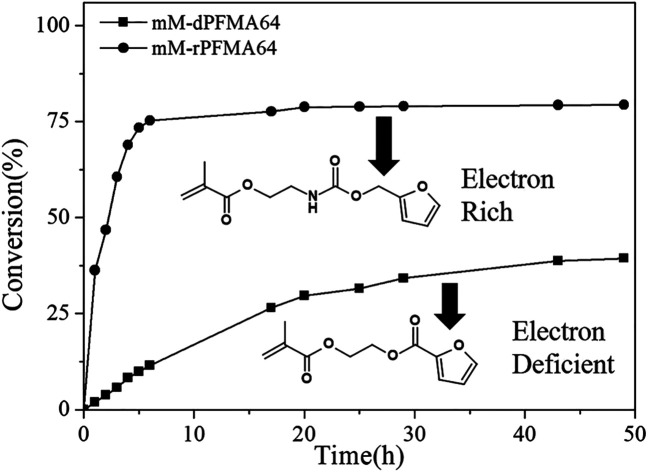

## 1. Introduction

Recently, there has been increased demand for smart polymeric materials^1,2^ that have a specific response to external stimuli like heat, pressure, pH, *etc.* Among these stimuli responsive polymers, self-healable polymers, which can recover their pristine properties after micro/macroscale physical damage, have generated tremendous interest owing to their durable natures. The self-healing efficiency can effectively increase the stability and lifetime of polymeric materials which are vulnerable to long-term chemical or physical fatigue.

To achieve the high efficiency of self-healing, there are two essential requirements; one is diffusion of molecules into crack areas and the other is interactions (or reactions) between the molecules. Firstly, self-healable molecules that induce self-healing properties should have enough mobility to fill the crack areas. Then, there should be interactions (or reactions) between the self-healable molecules that can help replace and/or recover the pristine physical and/or chemical properties of matrices. Many researches have tried to design various self-healing systems that can satisfy these two factors by embedding the self-healable structures into matrices such as microcapsules,^3–5^ microvascular networks,^6,7^ carbon nanotubes,^8,9^ and so on.^10^

Despite the highly efficient self-healing performance of the self-healable structures embedded systems, one of the major limitation is that the self-healing process is not inherently repeatable.^10^ Therefore, several other intrinsic self-healing methods have utilized reversible chemical reactions such as disulfide bond formation,^11,12^ the metal–ligand coordination reaction,^13^ hydrogen bonding^14,15^ and cycloaddition reaction, to achieve the multiple repetitions of the healing process.^16–20^ In such cases, repeated healing at the same damage site could be achieved without further any additional chemicals and/or healing agents. Among the reversible reactions, Diels–Alder (DA) reaction is one of the most commonly exploited chemical reaction used in various chemical syntheses and applications.^21–23^ This well understood [4 + 2] cycloaddition between a conjugated diene and dienophile could be easily used to form or cleave (retro Diels–Alder, rDA) by controlling the temperature without the need for any catalyst or other external stimuli. Applications of the DA reaction in self-healing materials have been widely studied due to the aforementioned advantages.^24–29^ However, many studies were focused on synthesizing new self-healable materials based on the DA reaction rather than investigating the influence of the reactivity of the DA reaction on self-healing efficiency. Therefore, herein, to study the influence of the reactivity of the DA reaction on self-healing efficiency, self-healable polymethacrylates having furan pendant groups with different electron densities were synthesized and characterized. Then, the DA reactivities of self-healable polymethacrylates with bismaleimide cross-linkers and the self-healing efficiencies of as-prepared DA polymer networks were examined by various experiments.

## 2. Materials and methods

### 2.1. Materials

Furfuryl alcohol (FA; 98%), poly(ethylene glycol) methyl ether methacrylate(PEGMMA; average Mn = 500, contains 100 ppm MEHQ and 200 ppm BHT as inhibitor), dibutyltin dilaurate (95%), 1,1′-(methylenedi-4,1-phenylene)bismaleimide (bM; 95%) and 2-hydroxyethyl methacrylate(HEMA; contains 250 ppm monomethyl ether hydroquinone as inhibitor, 97%) were purchased from Sigma-Aldrich and used as received. 2-Furoyl chloride (FC; 98%) and 2-isocyanato ethyl methacrylate (NCO-MA, stabilized with BHT; 98%) were purchased from Tokyo Chemical Industry and used as received. α,α′-Azobis(isobutyronitrile) (AIBN; 98%) was purchased from Tokyo Chemical Industry and recrystallized with ethanol before used. Methylene chloride (MC), *N*,*N*-dimethylformamide (DMF), tetrahydrofuran (THF), *n*-hexane, chloroform, diethyl ether and pyridine (99.5%) were purchased from Daejung Chemicals and used after dehydration with molecular sieves.

### 2.2. Synthesis of self-healing materials

#### 2.2.1. Synthesis of electron-deficient furan-functionalized monomer (dFMA)

FC (202 mmol, 26.48 g) diluted with MC (100 mL) was added to a stirred solution of HEMA (243 mmol, 31.65 g) and pyridine (243 mmol, 19.17 g) in 200 mL of MC at 0 °C. The reaction mixture was stirred at RT for 24 hours. Subsequently, the product was poured into separatory funnel with water and washed several times. The organic layer was then dried over anhydrous sodium sulfate and filtered. The product solution was evaporated under reduced pressure to remove the solvent. ^1^H NMR (400 Hz, CDCl_3_, ppm, *δ*): 7.61 (s, 1H), 7.21 (d, 1H) and 6.53 (d, 1H), 6.14 (s, 1H), 5.60 (s, 1H), 4.53 (t, 2H), 4.47 (t, 2H), 1.95 (s, 3H).

#### 2.2.2. Synthesis of electron-rich furan-functionalized monomer (rFMA)

NCO-MA (71 mmol, 10.98 g), FA (106 mmol, 10.44 g) and a few drops of dibutyltin dilaurate were dissolved in 200 mL of chloroform and stirred at 50 °C for 24 h. The product was then poured into a separatory funnel with water and washed several times. The organic layer was then dried over anhydrous sodium sulfate and filtered. The product solution was evaporated under reduced pressures to remove the solvent. ^1^H NMR (400 Hz, CDCl_3_, ppm, *δ*): 7.41 (s, 1H), 6.40 (d, 1H), 6.34 (d, 1H), 6.10 (s, 1H), 5.58 (s, 1H), 5.05 (s, 2H), 4.22 (t, 2H), 3.50 (t, 2H), 1.93 (s, 3H).

#### 2.2.3. Polymerization of poly(furoyl methacrylate)-co-poly(poly(ethylene glycol) methyl ether methacrylate) (dPFMA)

A series of poly(furoyl methacrylate)-co-poly(poly(ethylene glycol) methyl ether methacrylate) (dPFMA#s, where # represents the molar feed ratio of furoyl methacrylate and poly(ethylene glycol) methyl ether methacrylate) having different contents of furan side group were synthesized by adjusting the feed ratio of the monomers. The synthetic procedures for dPFMA64 are provided here as an example. dFMA (60 mmol, 13.44 g), PEGMA (40 mmol, 20.00 g) and AIBN (0.5 wt% of dFMA) in 334 mL of THF were taken in a three-necked round bottom flask. The mixture was stirred at reflux for 4 h and poured into *n*-hexane. The precipitant was filtered and dried.

#### 2.2.4. Polymerization of poly(furfuryl methacrylate)-co-poly(poly(ethylene glycol) methyl ether methacrylate) (rPFMA)

A series of poly(furfuryl methacrylate)-co-poly(poly(ethylene glycol) methyl ether methacrylate) (rPFMA#s, where # represents the molar feed ratio of furfuryl methacrylate and poly(ethylene glycol) methyl ether methacrylate) having different contents of furan side group were synthesized by adjusting the ratio of the monomers. The synthetic procedures for rPFMA64 are provided here as an example. rFMA (60 mmol, 15.20 g), PEGMA (40 mmol, 20.00 g) and AIBN (1 wt% of rFMA) in 352 mL of THF were taken in a three-necked round bottom flask. The mixture was stirred at reflux for 4 hours and then poured into diethyl ether. The precipitant was filtered and dried.

### 2.3. Self-healing test

#### 2.3.1. Preparation of cross-linked polymer films

dPFMA or rPFMA and bM cross-linker (furan/maleimide molar ratio = 1 : 1) were dissolved in DMF to prepare 10% solution. The solution was poured onto a silicon mold (70 mm × 12 mm) and dried in air at 120 °C for 24 h. The film was then heated in air at 60 °C for 24 h to prepare a cross-linked polymer film of bM-dPFMA# or bM-rPFMA#.

#### 2.3.2. Healing efficiency test

A rectangular test specimen (70 mm × 12 mm) obtained from the cross-linked polymer film preparation step was damaged by tapping with a 5 mm wide razor blade at RT. A 5 mm wide hole was made by full-penetration of the razor blade. Crack healing experiment was performed at 120 °C for 1 hour and 60 °C for 24 hours in air. Second crack was formed at the same area in which the first crack was formed, and the healing procedure was identical to the first crack.

### 2.4. Kinetic test

#### 2.4.1. Preparation of monofunctionalized maleimide (mM)

4-(2-Hydroxyethyl)-10-oxa-4-azatricyclo[5.2.1.02,6]dec-8-ene-3,5-dione (protected hydroxyethyl maleimide) was synthesized according to the literature.^30^ Purified protected hydroxyethyl maleimide was dissolved in DMF and stirred at 120 °C for 3 h. The solution was then poured into diethyl ether and filtered. Yellow powders were obtained after the removal of the solvent under reduced pressure. ^1^H NMR (400 Hz, DMSO-d_6_, ppm, *δ*): 7.01 (s, 2H), 3.47 (t, 2H), 3.39 (t, 2H).

#### 2.4.2. Kinetics

An equivalent molar mixture of dPFMA64 or rPFMA64 and mM was dissolved in THF and the solvent was removed by rotary evaporation under reduced pressure at 30 °C for 30 minutes. Then, a homogeneous mixture was obtained at specific time intervals at 60 °C and quickly dissolved in deuterated dimethyl sulfoxide and sealed in a glass tube to calculate the DA reaction conversion using ^1^H NMR.

### 2.5. Characterization

^1^H NMR spectra were measured using an Avance-400 spectrometer (Bruker). The *M*_w_ and polydispersity index were obtained by gel permeation chromatography (GPC, Viscotek). THF was used as the solvent and monodispersed polystyrene was used as the standards. Differential scanning calorimetry (DSC, DSC N-650, Scinco) was carried out at heating and cooling rated of 10 °C min^−1^ under nitrogen. Fourier-transform infrared spectra (FT-IR) were recorded in the attenuated total reflectance (ATR) mode over the frequency range 4000–400 cm^−1^ on an ALPHA-P (Bruker). Optical microscopy (OM) images were obtained with an optical microscope (DIMIS-M). The mechanical properties were obtained using a universal testing machine QM100S (Qmesys) at an elongation rate of 10 mm min^−1^ at RT.

## 3. Results and discussion

### 3.1. Preparation and characterization of furan-functionalized methacrylates

**Scheme 1:**
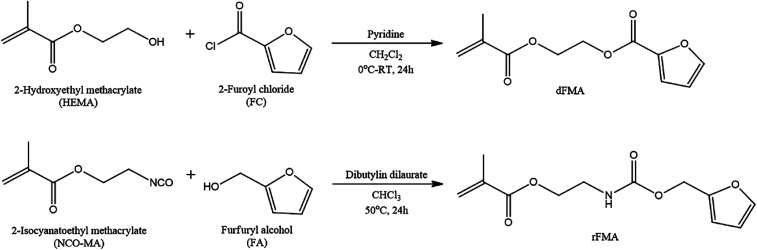
Synthesis of furan-functionalized monomers.

To examine the effect of furan pendant groups on self-healing efficiency, we designed two types of polymethacrylates derivatives having furan pendant groups with different electron densities. As shown in Scheme 1, the difference in electron density was controlled by specimens of adjacent functional groups of furan pendant groups. The carbonyl groups were selected as electron-withdrawing group. The monomer containing furan pendant group which is expected to have relatively low electron density due to the adjacent electron-withdrawing group, carbonyl group, was named dFMA. And the monomer containing furan pendant group with adjacent methylene group which is expected to have the relatively low electron-withdrawing ability compared with one of carbonyl group, was named rFMA. The difference of the electron density between dFMA and rFMA was clearly confirmed by the chemical shifts in the ^1^H NMR analyses, which will be further discussed in the later part. (In the name of each monomer, “d” means electron deficient and “r” means electron rich.).^31,32^

**Fig. 1:**
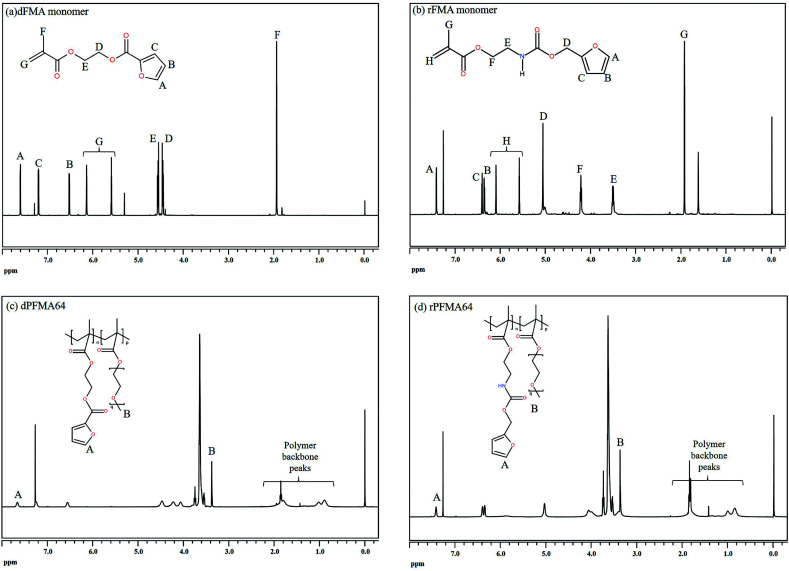
^1^H NMR spectra of (a) dFMA, (b) rFMA, (c) dPFMA64 and (d) rPFMA64.

dFMA was obtained from the reaction between HEMA and FC in the presence of pyridine and rFMA was obtained from the reaction between NCO-MA and FA in the presence of tin catalyst, respectively. After purification by washing with deionized water, each furan-functionalized methacrylates was characterized with ^1^H NMR (Fig. 1(a) and (b)).

The furan-functionalized methacrylates (dFMA and rFMA) were then polymerized with PEGMA as co-monomers *via* free radical polymerization. To enhance effect of the mobility of the polymer main chain on self-healing properties, PEGMA was selected as the co-monomer for the preparation of furan-functionalized polymethacrylates (dPFMA and rPFMA). The compositions of dPFMA and rPFMA were changed by controlling the molar feed ratios of dFMA and rFMA to PEGMA as 10/0 and 6/4, respectively. For dPFMA64 as an example, where 6 and 4 denote the molar ratios of dFMA and PEGMA in the feed ratio, the chemical structure was confirmed *via*^1^H NMR spectroscopy as shown in Fig. 1(c) and (d).

As shown in Fig. 1(a), the characteristic peaks at 7.62, 7.21 and 6.52 ppm are assigned to the protons of the furan pendant group in dFMA. After the polymerization (Fig. 1(c)), the disappearance of the methacrylate double bond peaks at 6.15 and 5.60 ppm in dFMA and the appearance of both the broad peaks of methacrylate backbone in the range 1.96–0.88 ppm and of the methyl group in PEGMA at 3.49 ppm indicate that the furan-functionalized polymethacrylates successfully were synthesized. In the same manner, all peaks of rFMA and rPFMA64 shown in Fig. 1(b) and (d) could be assigned and we could confirm all materials were successfully synthesized. As expected, in the case of rPFMA64, the proton peaks of the furan pendant group were shifted to up-field area at 7.42, 6.41 and 6.35 ppm compared to those of dPFMA64 at 7.62, 7.21, and 6.52 ppm due to the furan pendant group with relatively rich-electron density as shown in Fig. 1(c) and (d). Similar chemical shift for the protons peaks of furan pendant group were also shown in furan-functionalized methacrylates (Fig. 1(a) and (b)). This indicates that the electron density of furan pendant, which is considered to have an important role in determining the efficiency of self-healing, was successfully controlled by the adjacent carbonyl group of dFMA. The dFMA and rFMA contents in dPFMA and rPFMA were calculated by comparing the integral of the furan ring peak area (‘A’ peak in Fig. 1(c) and (d)) and the methyl group peak area (‘B’ peak in Fig. 1(c) and (d)).

The product ratio of rPFMA64 was different from that of dPFMA64 due to the difference in reactivities between the monomers, however, it was found that the difference was not critical enough to have a significant effect on the chemical or mechanical properties. The molecular weight and PDI values of the furan-functionalized polymethacrylates obtained in this study from GPC analysis were controlled to a similar level to exclude the effect of molecular weight on the efficiency of self-healing, as shown in Table 1.

**Table 1:** Characterization of the dPFMA and rPFMA series

Samples	Feed ratio [X^a^ : PEGMA]	Product ratio^b^ [X^a^ : PEGMA]	*M*_w_ c (×10^4^, RID)	PDI^c^	*T*_g_ (°C)^d^
dPFMA64	6 : 4	5.98 : 4.02	1.9	1.51	<−20
dPFMA100	10 : 0	10 : 0	3.1	1.81	41.76
rPFMA64	6 : 4	5.75 : 4.25	1.7	1.39	<−20
rPFMA100	10 : 0	10 : 0	3.1	1.94	55.14

a Furan-functionalized monomer (dFMA or rFMA).

b Composition of X *versus* PEGMA determined by ^1^H NMR.

c Determined by GPC using refractive index detector (RID) and calibrated with linear polystyrene standards (THF).

d DSC analyses were performed in range of −20 °C to 180 °C.

### 3.2. Thermo-reversible properties of furan-functionalized polymethacrylates

**Fig. 2:**
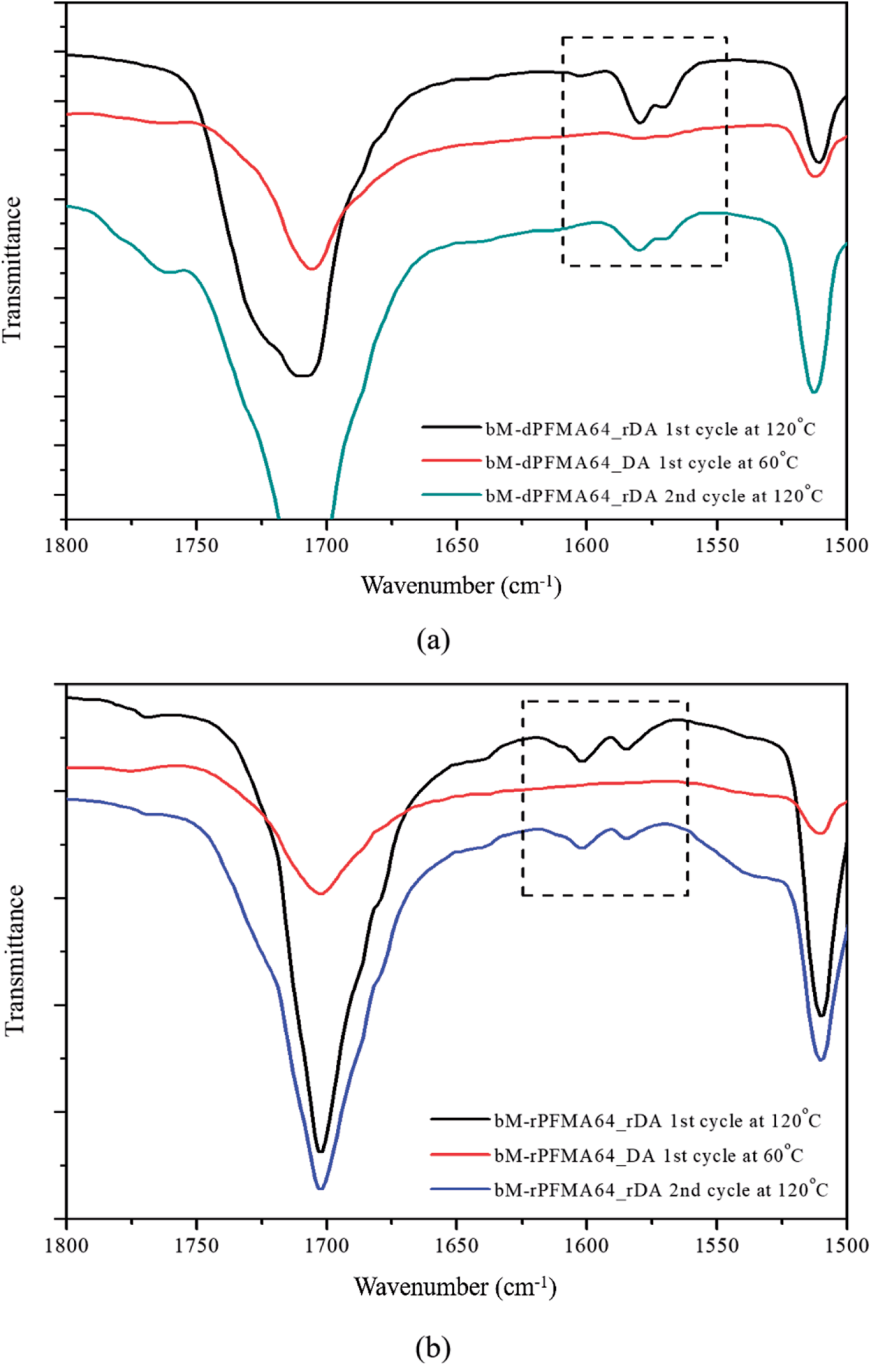
FT-IR spectra of (a) bM-dPFMA64 and (b) bM-rPFMA64.

Before the test of self-healing efficiency of furan-functionalized polymethacrylates, the thermally reversible Diels–Alder (DA) and retro Diels–Alder (rDA) reaction between furan-functionalized polymethacrylates and bismaleimide was confirmed using FT-IR experiments. First, we prepared the cross-linked polymer film (bM-dPFMA64) using dPFMA64 and 1,1′-(methylenedi-4,1-phenylene)bismaleimide (bM) as a cross-linker. An equivalent amount of dPFMA64 and bM in DMF was prepared and casted on a silicon mold. After the evaporation of the solvent, a free-standing cross-linked polymer film was obtained (bM-dPFMA64). As can be seen in Fig. 2(a), the absorption peaks at 1581 and 1569 cm^−1^ originating from the double bond of the furan pendant group in dPFMA64 are observed after the rDA reaction at 120 °C (black line). These peaks disappeared after thermal treatment for 24 h at 60 °C (red line) due to the [4 + 2] cycloaddition by the DA reaction, then reappeared upon thermal treatment at 120 °C for 1 h (green line). This indicates that the thermally reversible DA/rDA reactions between dPFMA64 and bM successfully occurred during the heat treatment processes. Similar phenomena were also observed in the film prepared with rPFMA64 and bM, bM-rPFMA64 (Fig. 2(b)). The absorption peaks at 1601 and 1585 cm^−1^ observed after heat treatment at 120 °C (black line) completely disappeared due to the DA reaction at 60 °C (red line) and reappeared as a result of the rDA reaction at 120 °C (blue line). However, from a comparison of the FT-IR spectra obtained after the heat treatment at 60 °C (red lines in Fig. 2(a) and (b)), it was found that there still remained unreacted furan pendant groups in the bM-dPFMA64 film, even though almost all the furan pendant groups in rPFMA64 participated in the DA reaction under the same heat treatment condition. This is possibly due to the difference in the reactivities of the furan pendant groups of rPFMA64 and dPFMA64, which will be further confirmed by kinetic studies, as reported in subsequent sections in this paper.

**Fig. 3:**
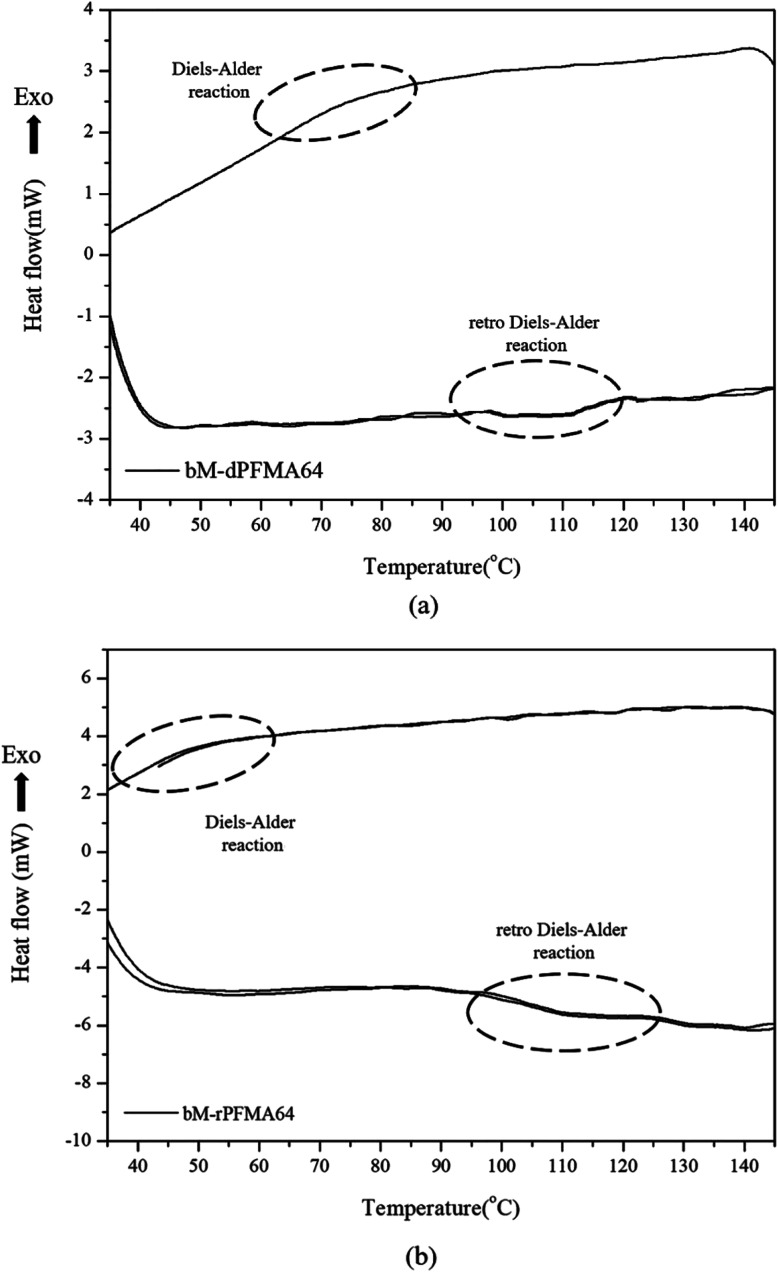
Repeated DSC curves of (a) bM-dPFMA64 and (b) bM-rPFMA64.

For a more detailed study of the thermal reversibility of DA/rDA reaction in bM-rPFMA64 and bM-dPFMA64 films, thermal analyses were performed using DSC. Fig. 3 shows that DSC results of twice repeated heating and cooling cycles ranging between 35 °C and 145 °C for both the bM-dPFMA64 and bM-rPFMA64 films. As shown in Fig. 3(a), the first heating scan showed a broad endothermic peak at about 110 °C, indicating the rDA reaction in the bM-dPFMA64 film. The broad exothermic peak below 80 °C in the cooling cycle indicates that the DA reaction between the furan and maleimide groups which were reformed by the rDA reaction in the heating scan was occurred in the bM-dPFMA64 film. In the second cycle, the same thermal behavior as that observed during the first cycle could be observed. The DSC profiles of bM-rPFMA64 also showed similar thermal behaviors during two cycles of repeated heating and cooling cycles (Fig. 3(b)). In this manner, we confirmed that the furan-functionalized polymethacrylates in this study can reversibly react with bM by heat treatment and concluded that it can be further applied as repeatable self-healing material based on the DA reaction.

### 3.3. Dependence of self-healing performance on polymer chain mobility

**Fig. 4:**
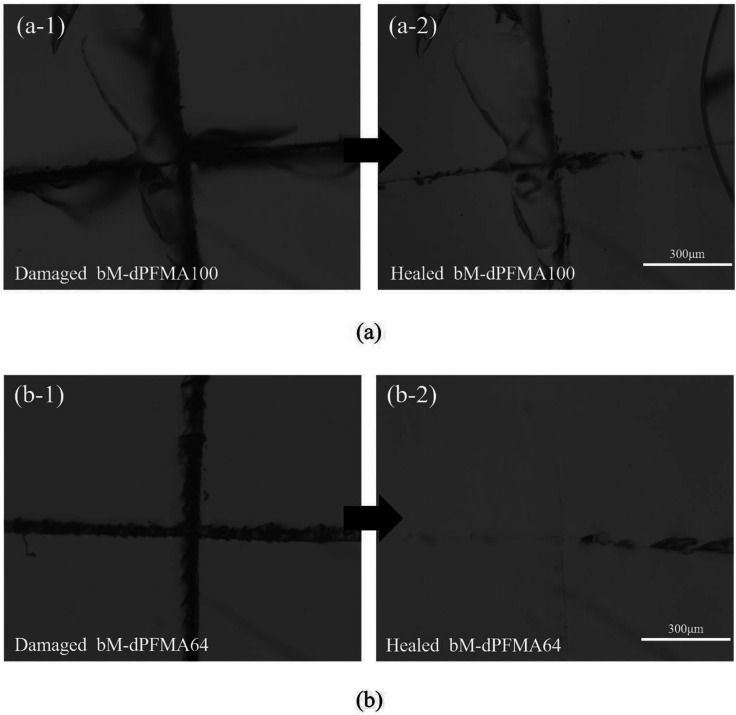
Optical microscopy images of damaged bM-dPFMA100 and bM-dPFMA64 ((a-1) and (b-1)) and those treated at 120 °C for 60 min and 60 °C for 24 h ((a-2) and (b-2)).

**Scheme 2:**
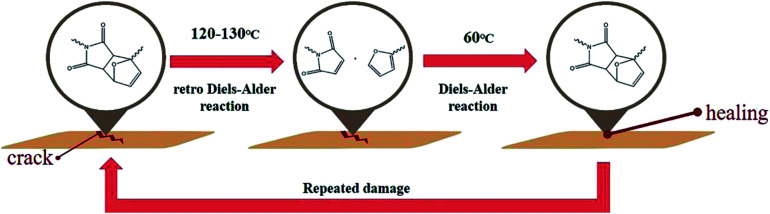
Self-healing process of the Diels–Alder based polymeric material.

Prior to the comparison of the self-healing efficiencies of dPFMA and rPFMA, we investigated the dependence of the thermally induced self-healing performance of dPFMAs on their compositions. The self-healing behavior was examined by optical microscopy (OM). Fig. 4 shows the OM images of the bM-dPFMAs films before (left) and after (right) the healing processes. The artificial cracks of width 20–40 μm on the film surface of bM-dPFMA100 and bM-dPFMA64 were made using a razor blade, as shown in Fig. 4(a-1) and (b-1). After healing process by heat treatments at 120 °C for 1 h and 60 °C for 24 h, the bM-dPFMAs films exhibited different healing performance depending on their compositions (Fig. 4(a-2) and (b-2)). While almost complete self-healing behavior could be observed in the bM-dPFMA64 films, the damaged bM-dPFMA100 film was either partially healed or not healed at all. Since it is already known that heat treatment at 120 °C is sufficient to induce the rDA reaction in the bM-dPFMA film from the previous FT-IR and DSC experiments, we expected that the difference in self-healing performance between the bM-dPFMA64 and bM-dPFMA100 films is originated from the intrinsic chain mobilities of dPFMAs. The mobility of polymer chains for dPFMA64 should be relatively high compared to that of dPFMA100 due to the introduction of PEGMA, which could be confirmed by the glass transition temperature of both dPFMA64 and dPFMA100 as shown in Table 1.^33^ In the case of the dM-dPFMA64 film, the dPFMA64 backbone chains cleaved by the rDA reaction upon heat treatment at 120 °C relatively easily penetrate the cracks and fill them up by forming of chain entanglements.^34^ However, a further decrease in the content of the furan pendant groups in the polymer backbone to increase the polymer chain mobility rather decreased the self-healing performance due to an insufficient amount of the DA reaction sites in the damaged area. Therefore, considering both self-healing ability and mechanical property, the bM-dPFMA64 film was chosen as a model for further investigation. For a comparison, bM-rPFMA64 was also selected due to its similarities. The overall healing process in this study is depicted in Scheme 2.

### 3.4. Self-healing efficiency

The self-healing behavior of bM-dPFMA64 and bM-rPFMA64 was studied by calculating the healing efficiency obtained through the measurement of the mechanical strength of the original, damaged, and healed films *via* UTM experiments. The healing efficiency was calculated from eqn (1)1
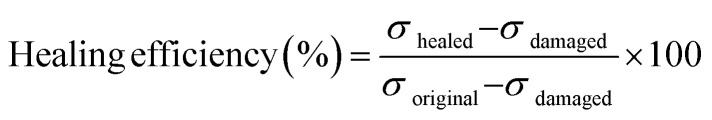
*σ* refers tensile strength that measured from UTM test.^35^

**Fig. 5:**
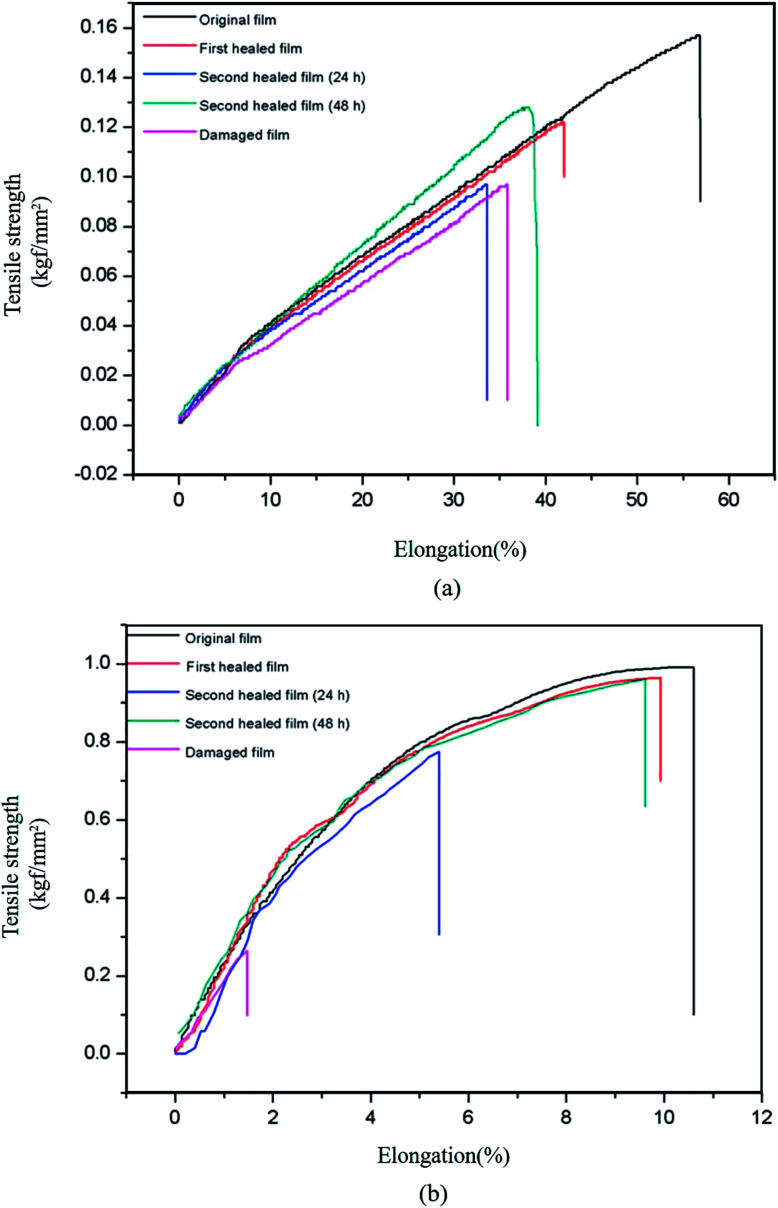
Stress–strain curves of original, damaged and healed samples of (a) bM-dPFMA64 and (b) bM-rPFMA64.

Fig. 5 shows the stress–strain curves of the original, damaged, and healed bM-dPFMA64 and bM-rPFMA64 films. Although the chemical structures of dPFMA64 and rPFMA64 are very similar, in the case of original films, bM-rPFMA64 film shows higher tensile strength and lower elongation at break compared with ones of bM-dPFMA64 film. It could be explained by the relatively high degree of crosslinking in bM-rPFMA64 film which is originated from the higher DA reaction efficiency as shown in Fig. 2. The healing processes consisted of heat treatment of the damaged film at 120 °C for 1 h and 60 °C for 24 h. First, in Fig. 5(a), the bM-dPFMA64 film showed efficiencies of 42.37% after the first healing process and 1.69% from the second healing process. On the other hand, the healing efficiencies of the bM-rPFMA64 film are higher than those of the bM-dPFMA64 film being 95.89% for the first and 69.86% for the second healing processes as shown in Fig. 5(b). It is considered that the types of the furan pendant groups have a major effect on the healing efficiency. Relatively high electron density of the furan pendant group in rPFMA64 compared to that of dPFMA64, which was previously confirmed in NMR experiments, induces higher reactivity with bM in the DA reaction under identical conditions, which in turn, has a positive effect on the self-healing efficiency of the bM-rPFMA64 film. This could be further supported by the dependence of healing efficiency on the DA reaction time. When the DA reaction time in the second healing process increased to 48 h to investigate the effect of time on self-healing, the healing efficiency in both the bM-dPFMA64 and bM-rPFMA64 films increased as the DA reaction time increased, as shown in Table 2. In particular, in the case of bM-dPFMA64, a significant difference in healing efficiency depending on time was observed at 1.69% for 24 hours and 52.54% for 48 hours. This could be considered in terms of the fact that the longer DA reaction time increases the possibility of contact between the furan pendant groups in the polymer backbones and bismaleimide molecules. However, the degree of increase in bM-rPFMA64 is much higher than that in the bM-dPFMA64 film, implying that the reactivity of the furan pendant group in rPFMA64 is higher than that in dPFMA64, where it originated from the difference of electron densities for the furan pendant groups. In the case of bM-rPFMA64, the hydrogen-bonding could also have the effect on the healing efficiency. Compared with the dPFMA64 containing ester bond adjacent to furan group, the rPFMA64 containing urethane bond adjacent to furan group can form the hydrogen-bonding with C=O of maleimide.^36^ Therefore, the combination effect between the electron density for furan pendant group and hydrogen-bonding could increase the reactivity of DA reaction of bM-rPFMA64, which in turn, induce the relatively higher healing efficiency.

**Table 2:** Healing efficiencies of bM-dPFMA64 and bM-rPFMA64

Samples	First healing	Second healing (24 h)	Second healing (48 h)
bM-dPFMA64	42.37%	1.69%	52.54%
bM-rPFMA64	95.89%	69.86%	94.52%

### 3.5. Kinetic analysis

**Fig. 6:**
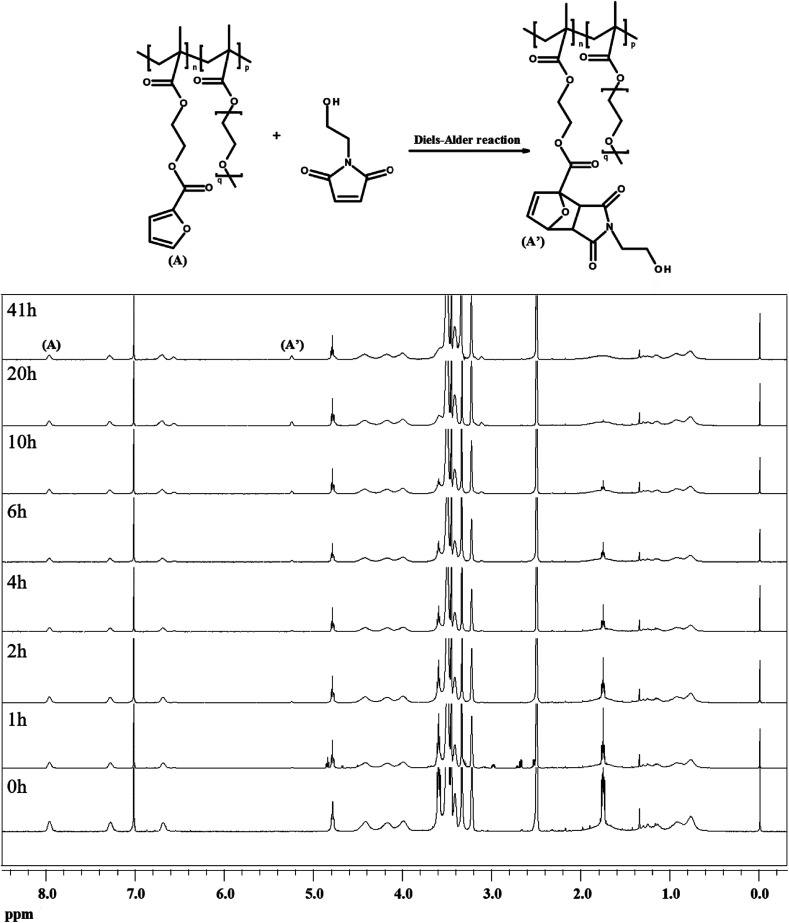
^1^H-NMR spectra of the mixture of dPFMA64 and mM kept at 60 °C for 0 to 41 h.

To investigate the dependence of the reactivity of furan pendant groups on electron density, an indirect kinetic study of the DA reaction between dPFMA64 and rPFMA64 with monofunctionalized maleimide (mM) was designed and monitored using ^1^H-NMR experiments. The mM instead of bM was used as a model compound to prevent intermolecular cross-linking reactions that can inhibit the monitoring of the ^1^H-NMR experiments. As shown in Fig. 6, the ratio between the integrated peak of proton (A) of the unreacted furan pendant group in dPFMA64 and proton (A′) of the reacted furan pendant group in the dPFMA64 mM adduct was calculated at different time intervals.

The same experiments were repeated using rPFMA64 and mM (data is not shown). The time-dependent reaction conversion of the DA adduct, *x*, can be calculated from eqn (2).2
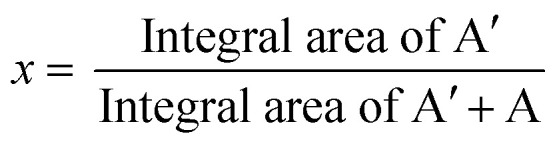


Since the DA reaction of dPFMA64 and mM follows second-order kinetics,^15^ the rate equation can be obtained from eqn (3).3
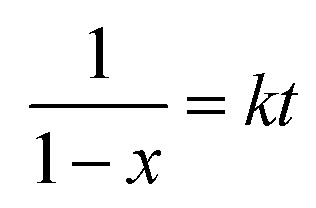


**Fig. 7:**
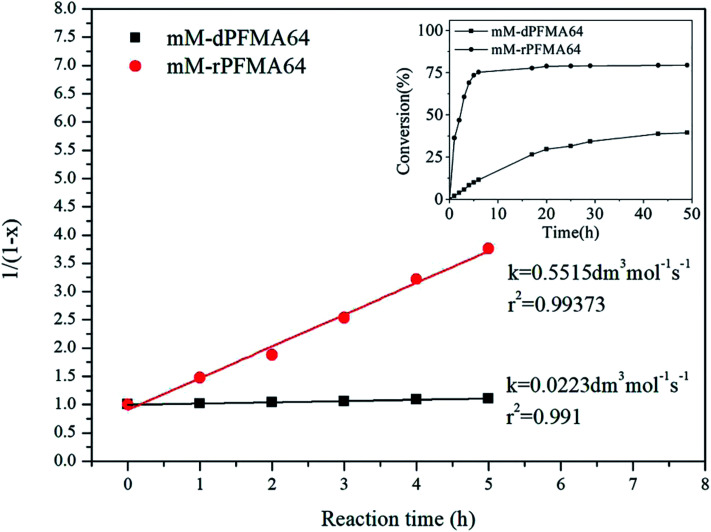
The second-order kinetic plot for the DA reaction between mM and dPFMA64/rPFMA64 at 60 °C. (inset: the conversion *versus* time plot of each samples).

The rate constant *k* for the DA reaction of dPFMA64 and rPFMA64 with mM are shown in Fig. 7. The *k* value of rPFMA64, 0.5515 dm^3^ mol^−1^ s^−1^, which was 24 times higher than that of dPFMA64, 0.0223 dm^3^ mol^−1^ s^−1^. Additionally, the reaction conversion of dPFMA64 and rPFMA64 with mM are shown in inset of Fig. 7. The DA reaction conversion for dPFMA64 was 31.5% at 24 h, while that of rPFMA64 was 78.9% for the same duration. From the above results, the rate and the conversion of DA reaction of rPFMA64 are significantly higher than those of dPFMA64. Since the DA reaction is normally a pericyclic reaction between the electron-rich diene and the electron-deficient dienophile, the electron-withdrawing carbonyl group adjacent to the furan pendant group of dPFMA can impede the DA reaction, which leads to the retarded healing efficiency of the bM-dPFMA64 film.

## 4. Conclusion

rPFMA64 and dPFMA64 that have different electron density of furan pendant group were synthesized through free-radical polymerization to clarify the dependence of electron density in furan groups on the efficiency of self-healing property. PEGMA was also introduced as a co-monomer to improve the mobility. The structures of all furan-functionalized polymethacrylates were characterized *via*^1^H-NMR. Then, cross-linked films were prepared by DA reaction of the furan-functionalized polymethacrylates and the bM. And FT-IR and DSC analysis confirmed that DA/rDA reaction were repeatedly caused by heat treatment. For the self-healing test, tensile strength measurement *via* UTM was performed to each state of films. As the result, bM-rPFMA64 film which has relatively high electron density in furan pendant group showed the healing efficiency of 95.89% at first healing cycle and 69.98% at second healing cycle. In addition, the healing efficiency was 94.52% when the DA reaction was carried out for 48 hours in second healing. These healing efficiencies were higher than those case of bM-dPFMA64 film. Lastly, the kinetic test using ^1^H-NMR was performed to interpret these results and it was confirmed that the DA conversion rate of mM-rPFMA64 rapidly increased in a shorter time than mM-dPFMA64 under the same conditions. And from these results, it could be concluded that the increase in the reactivity of the furan pendant groups leads to the improvement in the healing efficiency in the self-healing system.

## Conflicts of interest

There are no conflicts to declare.

## Supplementary Material
